# Which aspects of job determine satisfaction among pharmacists working in Saudi pharmacy settings?

**DOI:** 10.1371/journal.pone.0289587

**Published:** 2023-08-04

**Authors:** Md. Ashraful Islam, Atta Abbas Naqvi

**Affiliations:** 1 Department of Pharmacy Practice, College of Clinical Pharmacy, Imam Abdulrahman Bin Faisal University, Dammam, Saudi Arabia; 2 Department of Pharmacy Practice, School of Pharmacy, University of Reading, Whiteknights Campus, Reading, United Kingdom; University of Petra (UOP), JORDAN

## Abstract

**Objective:**

The aim of this study was to evaluate the impact of several employment-related aspects on overall job satisfaction among pharmacists working in Saudi pharmacy settings.

**Methods:**

A cross-sectional survey was conducted for a period of 1-month (December 2020) among pharmacists working in community pharmacies located in 3 cities of Saudi Arabia. Convenience sampling was employed, and the data was collected using the English version of Job Satisfaction Survey (JSS) questionnaire. The data was analyzed using IBM SPSS version 23. Descriptive statistics such as mean ($\bar{x}$) and 95% confidence interval range were used to report continuous data; frequency (%) and sample counts (N) were used to report categorical data. Bivariate analyses were conducted using chi square (χ^2^) test. A multiple linear regression model was formulated to report the employment aspects that determined overall job satisfaction of pharmacists. The study was approved by an ethics committee.

**Results:**

A total of 241 samples were analyzed. Less than a quarter of pharmacists (N = 54, 22.4%) were satisfied with their job. The overall job satisfaction score was 130.74 out of 199. The sub-scales for co-workers and communication had scores > 15.8 out of 24; subscale for operating conditions had score > 12.5 out of 20. The subscales for promotion and rewards had scores < 14 out of 24. The aspects of communication, fringe benefits and nature of work had the highest contribution towards overall job satisfaction. For a unit increase in score for communication, fringe benefits, and nature of work, the overall job satisfaction score increased by 0.204, 0.2, and 0.199, respectively.

**Conclusion:**

A very small number of pharmacists seemed satisfied with their job. Satisfaction with communication, nature of work and fringe benefits contributed the most toward overall job satisfaction. Results of this study could provide the means for human resource managers and organizational policy makers to delve into the determinants of satisfaction among pharmacists working in community settings.

## Introduction

Job satisfaction could be considered as an emotional attachment of an employee to the workplace [1, 2]. While pharmacists may feel satisfied practicing pharmacy, the profession could become stressful [2, 3]. Few notable characteristics of community pharmacy practice include increased workload, long work shift, etc. However stress management skills among community pharmacists could come handy in these situations [4]. A study reported that 67.2% of pharmacists in the US were satisfied with employment [3]. A study in Australia highlighted job dissatisfaction as a core reason for job turnover [5]. It was also reported that stress at the workplace is one of the most common reasons mentioned by pharmacists for quitting employment [6].

From a theoretical standpoint, the theory of Maslow’s hierarchy of needs has been used previously to assess nurses’ motivation [7]. According to the theory there are 5 levels of human needs and is usually presented as a pyramid with 5 levels. The bottom level contains basic needs such as food, shelter, clothing, etc. followed by safety that includes employment, income, health, etc. The third level entails love and belonging that may translate into family, connections, friendship, etc. The fourth level entails esteem that incorporates recognition, respect, freedom, etc. The final top-most level is self-actualization. The theory further states that an individual can only ascend higher levels of needs after achieving the need of the previous level [8].

A study conducted among pharmacists working in community and hospital pharmacies in Jordan highlighted that pharmacists working in the community pharmacies were less satisfied as compared to those working in the hospital pharmacies. In addition, prolong work hours, lack of incentives, etc. acted as determinants of job satisfaction [9]. In a study conducted among pharmacists associated with hospital settings of Riyadh city in Saudi Arabia, it was reported that most pharmacists were satisfied with their work. However, most of them had an intention to leave [10]. Other studies conducted in Saudi Arabia highlighted that hospital pharmacists considered recognition, career advancement opportunities, autonomy, etc., as motivators while pharmacy managers considered financial rewards as major a driver for motivation for pharmacists as compared to non-financial rewards [11, 12]. Whereas a study among community pharmacists working in Riyadh region highlighted that independent community pharmacists were slightly less satisfied [2]. The pharmacists mentioned longer work hours, difficulty in securing work leaves, low financial incentives as few determinants of dissatisfaction [2].

The COVID-19 pandemic further added to the healthcare burden on community pharmacies as they become the primary point of contact for addressing the healthcare needs of the public [13]. Since March 2, 2020, a total of 500,083 cases have been reported in Saudi Arabia. The number of patients recovered was 481,225 while 7963 deaths have been reported so far. All figures are reported at the time of this writing, i.e., July 10, 2021 [14]. The Saudi health authorities adopted the World Health Organization’s recommendations and implemented a lockdown in the country [15]. The community pharmacies in Saudi Arabia remained opened for the public with newly adopted health guidelines.

To date, there have been few studies conducted to document employment satisfaction in Saudi pharmacy settings [2, 10–12]. Of these, only two studies evaluated community pharmacists’ job satisfaction and were conducted in Riyadh region [2, 10]. Hence, data pertaining to job satisfaction of pharmacists in Eastern Region of Saudi Arabia is not available. Based on the available evidence from aforementioned studies, it was observed that pharmacists working in Saudi healthcare settings especially the community sector usually seemed satisfied however, they did have a sense of dissatisfaction. Several reasons have been reported for this dissatisfaction such as type of work, financial benefits, cooperation with others, recognition, etc. [2]. However, which of these employment aspects impact a pharmacist’s overall job satisfaction the most, has never been investigated before. In addition, the pandemic has also affected the usual working pattern of the pharmacies. These aspects are essential to document as they can affect employment satisfaction. Also, documenting these aspects may provide an understanding of the determinants that affect overall satisfaction. This data could help in review and revision of employment contracts of pharmacists at workplaces.

The documentation and understanding of these aspects is essential to devise human resource policies that increase the chances of employee satisfaction and retention. As of now, no study has been conducted in this region that evaluates the extent to which employment aspects such as monetary benefits, vacations, promotions, etc., affect overall satisfaction with the job.

## Methods

### Study objective

To address the research question, i.e., which aspects of employment determine overall job satisfaction among pharmacists working in Saudi pharmacy settings, this study was aimed to evaluate the impact of several humanistic and employment-related aspects on overall job satisfaction among pharmacists working in these settings. These aspects included pay and promotion, benefits aside from salary, any rewards offered, the operating conditions and nature of work. It also includes the overall work environment that consists of co-workers, quality of communication and supervision.

### Study design, duration, and venue

A cross-sectional survey was conducted for a month (December 2020) among pharmacists working in retail and hospital-based community pharmacies located in Dammam region, Saudi Arabia.

### Target segment and eligibility criteria

Pharmacists working in community settings were the focus of this study. The inclusion criteria included, 1) all male and female pharmacists, 2) with a bachelor’s degree or post-graduation in pharmacy, 3) registered with the official regulatory body, and 4) currently practicing in retail and hospital-based community pharmacies. This population segment was invited to participate in the survey. The exclusion criteria were as follows: 1) Pharmacists working in in-patient pharmacies, and 2) pharmacists who were pharmacy owners. This segment was not included in the study. Pharmacists who were involved in in-patient pharmacies were not the focus of this work, and pharmacist who owned the pharmacies were self-employed.

### Sampling process

We collected data from pharmacies located in the cities of Dammam, Khobar, and Dhahran in Dammam Region. These cities are located in the Eastern Province of Saudi Arabia. The cities were divided hypothetically into 5 geographic regions, i.e., North, South, East, West, and Central. Convenient sampling was then applied at the level of each hypothetical geographic region, and the data were collected from pharmacy settings. According to the literature, a total of 24,395 pharmacy personnel work in Saudi Arabia out of which 4,531 are local while 19,864 are non-Saudis. A total of 8,419 pharmacists work in community pharmacy settings across the country. Besides, 3,738 pharmacists work in the Eastern Province of Saudi Arabia in all settings out of which 622 are locals while the rest are non-Saudis [16].

As per the figures from the 2021 statistics report of Saudi Ministry of Health, the total number of pharmacists working in the Ministry’s primary care centers in Eastern Province were 27 out of which 9 were males while 18 were females. A total of 2,401 (1,930 males, 471 females) pharmacists worked in private health sector in Eastern Province. Out of this, 565 (200 males and 365 females) were locals while 1,836 (1,730 males and 106 females) were non-Saudis [17]. The data pertaining to the number of pharmacists working in the private and public healthcare sector as well as community pharmacies specific to these three cities was not available. Therefore, we considered the pharmacists working in the Eastern Province, i.e., 3,738 as our target population.

Raosoft online based sample size calculator was used to calculate the sample size from the known population size of 3738 pharmacists [18]. Allowing for 95% confidence level and 7% marginal error, the recommended number of samples were 187. Data were collected during the pandemic period so that it was required to consider non-response rate. The formula by Sakpal (2010) was used to adjust sample size [19]. The adjusted sample size formula is:

$$n_{1}=n/(1-e)$$

Where *n* is the required sample size as per formula, *n*_*1*_ is adjusted sample size and, *e* is the potential missing or unintended error of the samples. We considered a 20% non-response rate, then the adjusted sample size was calculated and was 233.75. Finally, we targeted to collect 250 samples from different settings.

### Research instrument and data collection

A valid and pretested instrument coined as the ‘Job Satisfaction Survey (JSS)’ was used with permission from the developer [20]. The reason to use this scale was that it measured all of the aspects within employment along with overall job satisfaction. The JSS is a 36-item scale that not only measures overall job satisfaction but also measures satisfaction with employment aspects such as pay, promotion, supervision, benefits, rewards, operating condition, nature of work, co-workers, supervision, and communication. We used the English version of the scale available from the developer. The JSS is copyright © 1994, Paul E. Spector, All rights reserved. Reliability of the scale was assessed as a part of this study.

A demographic section was added that contained questions related to age, gender, workplace, monthly income, etc. It was designated as section 1 while the JSS was included as section 2. A consent form was added before section 1. The researchers visited the pharmacies in person and explained the study objectives to the pharmacists. The nature of consent was implied, i.e., participants could indicate their consent by asking for questionnaire. After obtaining implied consent from the participants, the questionnaire was provided to them. The participants filled in their responses and returned the questionnaire. The whole process took an average of 10 minutes. In some cases, the participants preferred the researchers to read the questions to them. They provided their response verbally and the investigators documented their answers. On the other hand, some respondents asked the researchers to pick up their filled questionnaire later. In that case, the filled questionnaire was picked up at a mutually agreed time.

### Data management

The data was analyzed using IBM SPSS version 23. Descriptive statistics such as mean ($\bar{x}$) and standard deviation or median (M) and interquartile range (IQR) were used to report the data depending upon distribution. Besides, 95% confidence interval range were also used for expressing continuous data. In addition, the minimum and maximum values were also reported. Frequency (%) and sample counts (N) were used to report categorical data. The independent variables (IV) considered were pharmacists’ demographic variables and work-related variables. The demographic variables were age, gender, nationality, marital status, and monthly income. The work-related variables were type of workplace, work experience, daily work hours, work overtime, and patients attended in daily shift. The dependent variables (DV) considered were the scores obtained for overall job satisfaction and each subscale.

The data pertaining to the JSS and subscale scores was continuous in nature. Besides, the score was also categorized into three categories, satisfied, ambivalent, and dissatisfied, according to scoring instructions [20]. Crosstabulation and chi square (χ^2^) test was used to assess the association between IV and DV by utilizing the categorical data for DV. Further, a multiple linear regression model was formulated that was adjusted for pharmacists’ demographics (IV). This model also included continuous data for scores obtained from the 9 subscales as DV and assessed their contribution towards overall satisfaction score. A total of nine (N = 9) completely missing surveys were cleaned which were detected using informal method [21].

### Ethics approval and consent

All participants were briefed about the research and were invited to participate. An implied consent was sought from the respondents. The consent form was provided to the participants at first. Those who agreed to participate indicated their consent in an implied manner. This was done by asking the researchers for the questionnaire. If a participant asked for the questionnaire after study briefing and reading the consent form, this meant that they agreed to participate in the survey. This method was utilized to minimize the risk of identification at the workplace. The study was subjected to review and was granted approval by the Institutional Review Board of Imam Abdulrahman Bin Faisal University through an expedited review (IRB– 2020–05–347).

## Results

After cleaning of completely missing surveys, 241 surveys were analyzed. The mean age of pharmacists was 32.72 ± 6.2 years. Most pharmacists were male (N = 168, 69.7%), and married (N = 175, 72.6%). Most pharmacists (N = 101, 41.9%) had a monthly income above SAR 10,000. Slightly more than half of the sample included Saudi pharmacists (N = 137, 56.8%) while rest were non-Saudi pharmacists. In addition, most Saudi pharmacists (N = 112) worked in hospital-based pharmacies while most non-Saudi pharmacists (N = 84) worked in retail pharmacies.

The mean years of work experience was 8.07 ± 5.96. The majority worked between 8–10 hours in a daily work shift (N = 219, 90.9%). Most pharmacists (N = 152, 63.1%) attended to more than 50 patients in a daily work shift. Few pharmacists had to work overtime (N = 94, 39%) and median hours of overtime was 4 (IQR = 8). The details are presented in Table 1.

**Table 1:** Background characteristics of the study variables (N = 241).

Characteristics	Frequency (N)	Percentage (%)
**Age in years**		
Age up to 30 years	108	44.8
Age more than 30 years	133	55.2
**Gender**		
Male	168	69.7
Female	73	30.3
**Nationality**		
Non-Saudi	104	43.2
Saudi	137	56.8
**Marital status**		
Single	66	27.4
Married	175	72.6
**Workplace**		
Hospital-based pharmacy	132	54.8
Retail pharmacy	109	45.2
Monthly income in SAR (USD)*		
< SAR 3,500, i.e., < USD 933.2	2	0.8
SAR 3,500–5,000, i.e., USD 933.2–1333.15	30	12.4
> SAR 5,000 < 7,500, i.e., USD > 1333.15 < 1999.72	67	27.8
> SAR 7,500 < 10,000, i.e., USD > 1999.72 < 2666.3	41	17
> SAR 10,000, i.e., USD > 2666.3	101	41.9
**Work experience in years**		
< 10	184	76.3
> 10	57	23.7
**Daily work hours**		
Less than 8 hours	10	4.1
Between 8 to 10 hours	219	90.9
More than 12 hours	12	5
**Work overtime**		
No	147	61
Yes	94	39
**Patients attended in daily work shift**		
15–20 patients	15	6.2
21–30 patients	24	10
30–50 patients	50	20.7
More than 50 patients	152	63.1

*1 USD equals SAR 3.75 at the time of this writing

The overall job satisfaction score was 130.74 out of a maximum possible score of 199. The sub-scales for co-workers and communication had scores > 15.8 out of a maximum possible score of 24 while the subscale for operating conditions had score > 12.5 out of 20. The subscales for promotion and rewards had scores <14 out of 24. The scores with 95% CI for all subscales and overall satisfaction along with reliability coefficient are reported in Table 2.

**Table 2:** Job satisfaction score and reliability analysis for different subscales and overall scale.

Subscale	α	Score	Lower	Upper	Minimum	Maximum
Pay	0.84	14.13	13.63	14.64	4	24
Promotion	0.73	13.95	13.49	14.42	4	24
Supervision	0.79	15.71	15.2	16.22	3	24
Fringe benefits	0.69	14.02	13.54	14.51	4	24
Contingent rewards	0.84	13.02	12.54	13.5	4	24
Operating conditions	0.42	12.68	12.29	13.06	6	20
Co-workers	0.31	15.88	15.37	16.38	2	24
Nature of work	0.44	15.74	15.19	16.29	2	24
Communication	0.84	15.88	15.33	16.42	4	24
Overall satisfaction	0.62	130.74	127.97	133.52	74	199

α = Cronbach’s alpha

The results for the distribution of job satisfaction based on demographic characteristics of the pharmacists is presented in Table 3. None of the demographic variables was significantly associated with overall job satisfaction (p > 0.05).

**Table 3:** Distribution of participants based on overall job satisfaction (N = 241).

Characteristics	Overall job satisfaction (N) (%)		
	Satisfied (N = 54, 22.4%)	Ambivalent (N = 161, 66.8%)	Dissatisfied (N = 26, 10.8%)
**Age (years)**			
≤ 30 years	28 (25.9)	69 (63.9)	11 (10.2)
> 30 years	26 (19.5)	92 (69.2)	15 (11.3)
**Gender**			
Male	40 (23.8)	113 (67.3)	15 (8.9)
Female	14 (19.2)	48 (65.8)	11 (15.1)
**Nationality**			
Saudi	32 (23.4)	88 (64.2)	17 (12.4)
Non-Saudi	22 (21.2)	73 (70.2)	9 (8.7)
**Marital status**			
Single	15 (22.7)	44 (66.7)	7 (10.6)
Married	39 (22.3)	117 (66.9)	19 (10.9)
**Workplace**			
Hospital-based	31 (23.5)	84 (63.6)	17 (12.9)
Retail	23 (21.1)	77 (70.6)	9 (8.3)
**Monthly income in SAR (USD)** *			
≤ SAR 5000, i.e., ≤ USD 1333.15	3 (9.4)	25 (78.1)	4 (12.5)
> SAR 5000, i.e., > USD 1333.15	51 (24.9)	136 (65.1)	22 (10.5)
**Work experience in years**			
≤ 10 years	39 (21.2)	124 (67.4)	21 (11.4)
> 10 years	15 (26.3)	37 (64.9)	5 (8.8)
**Daily work hours†**			
8 to 10	49 (22.4)	146 (66.7)	24 (11)
Other	5 (22.7)	15 (68.2)	2 (9.1)
**Work overtime**			
No	36 (24.5)	95 (64.6)	16 (10.9)
Yes	18 (19.1)	66 (70.2)	10 (10.6)
**Patients attended in daily work shift**			
≤ 30	6 (15.4)	30 (76.9)	3 (7.7)
> 30	48 (23.8)	131 (64.9)	23 (11.4)

*1 USD equals SAR 3.75 at the time of this writing, †Others denote less than 8 hours or more than 10 hours

The cross-tabulation analyses between demographic characteristics and subscales were conducted. The cross tabulation between income of pharmacist and satisfaction classification for the subscale of supervision was significant (p < 0.05). A larger proportion of pharmacists who had a monthly income of more than SAR 5000 that is equivalent to USD 1333.5, seemed satisfied with supervision (53.1%), as compared to those pharmacists with an income less than SAR 5000, i.e., USD 1333.5, i.e., (25%). The results are tabulated in Table 4.

**Table 4:** Distribution of job satisfaction based on subscales of pay, promotion, and supervision (n = 241).

Variables	Pay (N, %)			Promotion (N, %)			Supervision (N & %)		
	Satisfied (n = 80, 33.2%)	Ambivalent (n = 80, 33.2%)	Dissatisfied (n = 81, 33.6%)	Satisfied (n = 82, 34%)	Satisfied (n = 119, 49.4%)	Satisfied (n = 119, 49.4%)	Satisfied (n = 119, 49.4%)	Ambivalent (n = 73, 30.3%)	Dissatisfied (n = 49, 20.3%)
**Age (years)**									
≤ 30 years	42 (38.9%)	33 (30.6%)	33 (30.6%)	40 (37%)	37 (34.3%)	31 (28.7%)	57 (52.8%)	31 (28.7%)	20 (18.5%)
> 30 years	38 (28.6%)	47 (35.3%)	48 (36.1%)	42 (31.6%)	44 (33.1%)	47 (35.3%)	62 (46.6%)	42 (31.6%)	29 (21.8%)
**Gender**									
Male	56 (33.3%)	57 (33.9%)	55 (32.7%)	53 (31.5%)	58 (34.5%)	57 (33.9%)	79 (47%)	54 (32.1%)	35 (20.8%)
Female	24 (32.9%)	23 (31.5%)	26 (35.6%)	29 (39.7%)	23 (31.5%)	21 (28.8%)	40 (54.8%)	19 (26%)	14 (19.2%)
**Nationality**									
Saudi	48 (35%)	42 (30.7%)	47 (34.3%)	54 (39.4%)	41 (29.9%)	42 (30.7%)	73 (53.3%)	36 (26.3%)	28 (20.4%)
Non-Saudi	32 (30.8%)	38 (36.5%)	34 (32.7%)	28 (26.9%)	40 (38.5%)	36 (34.6%)	46 (44.2%)	37 (35.6%)	21 (20.2%)
**Marital status**									
Single	23 (34.8%)	22 (33.3%)	21 (31.8%)	21 (31.8%)	24 (36.4%)	21 (31.8%)	32 (48.5%)	22 (33.3%)	12 (18.2%)
Married	57 (32.6%)	58 (33.1%)	60 (34.3%)	61 (34.9%)	57 (32.6%)	57 (32.6%)	87 (49.7%)	51 (29.1%)	37 (21.1%)
**Workplace**									
Hospital-based	43 (32.6%)	42 (31.8%)	47 (35.6%)	49 (37.1%)	38 (28.8%)	45 (34.1%)	68 (51.5%)	36 (27.3%)	28 (21.2%)
Retail	37 (33.9%)	38 (34.9%)	34 (31.2%)	33 (30.3%)	43 (39.4%)	33 (30.3%)	51 (46.8%)	37 (33.9%)	21 (19.3%)
**Monthly income in SAR (USD)** * **‡**									
≤ SAR 5000, i.e., ≤ USD 1333.15	8 (25%)	14 (43.8%)	10 (31.3%)	7 (21.9%)	11 (34.4%)	14 (43.8%)	8 (25%)	16 (50%)	8 (25%)
> SAR 5000, i.e., > USD 1333.15	72 (34.4%)	66 (31.6%)	71 (34%)	75 (35.9%)	70 (33.5%)	64 (30.6%)	111 (53.1%)	57 (27.3%)	41 (19.6%)
**Work experience in years**									
≤ 10 years	60 (32.6%)	60 (32.6%)	64 (34.8%)	59 (32.1%)	62 (33.7%)	63 (34.2%)	92 (50%)	54 (29.3%)	38 (20.7%)
> 10 years	20 (35.1%)	20 (35.1%)	17 (29.8%)	23 (40.4%)	19 (33.3%)	15 (26.3%)	27 (47.4%)	19 (33.3%)	11 (19.3%)
**Daily work hours†**									
8 to 10	72 (32.9%)	72 (32.9%)	75 (34.2%)	72 (32.9%)	76 (34.7%)	71 (32.4%)	106 (48.4%)	65 (29.7%)	48 (21.9%)
Other	8 (36.4%)	8 (36.4%)	6 (27.3%)	10 (45.5%)	5 (22.7%)	7 (31.8%)	13 (59.1%)	8 (36.4%)	1 (4.5%)
**Work overtime**									
No	48 (32.7%)	43 (29.3%)	56 (38.1%)	55 (37.4%)	42 (28.6%)	50 (34%)	77 (52.4%)	40 (27.2%)	30 (20.4%)
Yes	32 (34%)	37 (39.4%)	25 (26.6%)	27 (28.7%)	39 (41.5%)	28 (29.8%)	42 (44.7%)	33 (35.1%)	19 (20.2%)
**Number of patients attended in daily work shift**									
≤ 30	14 (35.9%)	12 (30.8%)	13 (33.3%)	14 (35.9%)	12 (30.8%)	13 (33.3%)	21 (53.8%)	12 (30.8%)	6 (15.4%)
> 30	66 (32.7%)	68 (33.7%)	68 (33.7%)	68 (33.7%)	69 (34.2%)	65 (32.2%)	98 (48.5%)	61 (30.2%)	43 (21.3%)

*1 USD equals SAR 3.75 at the time of this writing

†Others denote less than 8 hours or more than 10 hours

‡Significant (p<0.05) for supervision subscale using Chi square (χ^2^) test

Besides, the cross tabulation between age of pharmacist and satisfaction classification for the subscale of operating condition was significant (p < 0.05). A larger proportion of pharmacists who were above 30 years seemed dissatisfied (50.4%) and satisfied (22.6%) with operating conditions, as compared to pharmacists aged 30 years or less, i.e., (43.5%) and (13.9%), respectively. The results are tabulated in Table 5.

**Table 5:** Distribution of job satisfaction based on subscales of contingent rewards, fringe benefits and operating conditions (n = 241).

Variables	Contingent rewards (N, %)			Fringe benefits (N, %)			Operating conditions (N & %)		
	Satisfied (n = 61, 25.3%)	Ambivalent (n = 68, 28.2%)	Dissatisfied (n = 112, 46.5%)	Satisfied (n = 86, 35.7%)	Ambivalent (n = 79, 32.8%)	Dissatisfied (n = 76, 31.5%)	Satisfied (n = 45, 18.7%)	Ambivalent (n = 82, 34.0%)	Dissatisfied (n = 114, 47.3%)
**Age (years)‡**									
≤ 30 years	27 (25%)	32 (29.6%)	49 (45.4%)	39 (36.1%)	37 (34.3%)	32 (29.6%)	15 (13.9%)	46 (42.6%)	47 (43.5%)
> 30 years	34 (25.6%)	36 (27.1%)	63 (47.4%)	47 (35.3%)	42 (31.6%)	44 (33.1%)	30 (22.6%)	36 (27.1%)	67 (50.4%)
**Gender**									
Male	42 (25%)	50 (29.8%)	76 (45.2%)	67 (39.9%)	52 (31%)	49 (29.2%)	30 (17.9%)	58 (34.5%)	80 (47.6%)
Female	19 (26.0%)	18 (24.7%)	36 (49.3%)	29 (39.7%)	19 (26%)	27 (37%)	15 (20.5%)	24 (32.9%)	34 (46.6%)
**Nationality**									
Saudi	33 (24.1%)	36 (26.3%)	68 (49.6%)	54 (39.4%)	50 (36.5%)	46 (33.6%)	31 (22.6%)	42 (30.7%)	64 (46.7%)
Non-Saudi	28 (26.9%)	32 (30.8%)	44 (42.3%)	36 (34.6%)	33 (31.7%)	35 (33.7%)	14 (13.5%)	40 (38.5%)	50 (48.1%)
**Marital status**									
Single	16 (24.2%)	18 (27.3%)	32 (48.5%)	18 (27.3%)	29 (43.9%)	19 (28.8%)	6 (9.1%)	24 (36.4%)	36 (54.5%)
Married	45 (25.7%)	50 (28.6%)	80 (45.7%)	68 (38.9%)	50 (28.6%)	57 (32.6%)	39 (22.3%)	58 (33.1%)	78 (44.6%)
**Workplace**									
Hospital-based	34 (25.8%)	35 (26.5%)	63 (47.7%)	47 (35.6%)	41 (31.1%)	44 (33.3%)	28 (21.2%)	40 (30.3%)	64 (48.5%)
Retail	27 (24.8%)	33 (30.3%)	49 (45%)	39 (35.8%)	38 (34.9%)	32 (29.4%)	17 (15.6%)	42 (38.5%)	50 (45.9%)
Monthly income in SAR (USD)*									
≤ SAR 5000, i.e., ≤ USD 1333.15	7 (21.9%)	10 (31.3%)	15 (46.9%)	6 (18.8%)	13 (40.6%)	13 (40.6%)	4 (12.5%)	11 (34.4%)	17 (53.1%)
> SAR 5000, i.e., > USD 1333.15	54 (25.8%)	58 (27.8%)	97 (46.4%)	80 (38.3%)	66 (31.6%)	63 (30.1%)	41 (19.6%)	71 (34.0%)	97 (46.4%)
**Work experience in years**									
≤ 10 years	44 (23.9%)	55 (29.9%)	85 (46.2%)	63 (34.2%)	60 (32.6%)	61 (33.2%)	32 (17.4%)	66 (35.9%)	86 (46.7%)
> 10 years	17 (29.8%)	13 (22.8%)	27 (47.4%)	23 (40.4%)	19 (33.3%)	15 (26.3%)	13 (22.8%)	16 (28.1%)	28 (49.1%)
**Daily work hours†**									
8 to 10	55 (25.1%)	62 (28.3%)	102 (46.6%)	80 (36.5%)	70 (32.0%)	69 (31.5%)	41 (18.7%)	77 (35.2%)	101 (46.1%)
Other	6 (27.3%)	6 (27.3%)	10 (45.5%)	6 (27.3%)	9 (40.9%)	7 (31.8%)	4 (18.2%)	5 (22.7%)	13 (59.1%)
**Work overtime**									
No	39 (26.5%)	45 (30.6%)	63 (42.9%)	52 (35.4%)	44 (29.9%)	51 (34.7%)	28 (19%)	50 (34%)	69 (46.9%)
Yes	22 (23.4%)	23 (24.5%)	49 (52.1%)	34 (36.2%)	35 (37.2%)	25 (26.6%)	17 (18.1%)	32 (34%)	45 (47.9%)
**Number of patients attended in daily work shift**									
≤ 30	6 (15.4%)	14 (35.9%)	19 (48.7%)	12 (30.8%)	17 (43.6%)	10 (25.6%)	6 (15.4%)	11 (28.2%)	22 (56.4%)
> 30	55 (27.2%)	54 (26.7%)	93 (46.0%)	74 (36.6%)	62 (30.7%)	66 (32.7%)	39 (19.3%)	71 (35.1%)	92 (45.5%)

*1 USD equals SAR 3.75 at the time of this writing

†Others denote less than 8 hours or more than 10 hours

‡Significant (p<0.05) for operating condition subscale using Chi square (χ^2^) test

Further, the cross tabulation between number of patients attended in daily work shift and satisfaction classification for the subscale of nature of work was significant (p < 0.05). A larger proportion (53.5%) of pharmacists who attended > 30 patients in a daily shift seemed satisfied with the nature of work as compared to those (35.9%) who attended < 30 patients in a daily shift. The results are presented in Table 6.

**Table 6:** Distribution of job satisfaction based on subscales of coworkers, nature of work and communication (n = 241).

Variables	Coworkers (N, %)			Nature of work (N, %)			Communication (N & %)		
	Satisfied (n = 134, 55.6%)	Ambivalent (n = 58, 24.1%)	Dissatisfied (n = 49, 20.3%)	Satisfied (n = 122, 50.6%)	Ambivalent (n = 68, 28.2%)	Dissatisfied (n = 51, 21.2%)	Satisfied (n = 120, 49.8%)	Ambivalent (n = 68, 28.2%)	Dissatisfied (n = 53, 22.0%)
**Age (years)**									
≤ 30 years	59 (54.6%)	27 (25%)	22 (20.4%)	54 (50%)	32 (29.6%)	22 (20.4%)	54 (50%)	26 (24.1%)	28 (25.9%)
> 30 years	75 (56.4%)	31 (23.3%)	27 (20.3%)	68 (51.1%)	36 (27.1%)	29 (21.8%)	66 (49.6%)	42 (31.6%)	25 (18.8%)
**Gender**									
Male	98 (58.3%)	39 (23.2%)	31 (18.5%)	83 (49.4%)	49 (29.2%)	36 (21.4%)	84 (50%)	47 (28%)	37 (22%)
Female	36 (49.3%)	19 (26%)	18 (24.7%)	39 (53.4%)	19 (26%)	15 (20.5%)	36 (49.3%)	21 (28.8%)	16 (21.9%)
**Nationality**									
Saudi	73 (53.3%)	29 (21.2%)	35 (25.5%)	67 (48.9%)	45 (32.8%)	25 (18.2%)	66 (48.2%)	42 (30.7%)	29 (21.2%)
Non-Saudi	61 (58.7%)	29 (27.9%)	14 (13.5%)	55 (52.9%)	23 (22.1%)	26 (25%)	54 (51.9%)	26 (25%)	24 (23.1%)
**Marital status**									
Single	37 (56.1%)	13 (19.7%)	16 (24.2%)	35 (53%)	19 (28.8%)	12 (18.2%)	33 (50%)	16 (24.2%)	17 (25.8%)
Married	97 (55.4%)	45 (25.7%)	33 (18.9%)	87 (49.7%)	49 (28%)	39 (22.3%)	87 (49.7%)	52 (29.7%)	36 (20.6%)
**Workplace**									
Hospital-based	66 (50.0%)	33 (25%)	33 (25%)	68 (51.5%)	43 (32.6%)	21 (15.9%)	62 (47%)	36 (27.3%)	34 (25.8%)
Retail	68 (62.4%)	25 (22.9%)	16 (14.7%)	54 (49.5%)	25 (22.9%)	30 (27.5%)	58 (53.2%)	32 (29.4%)	19 (17.4%)
**Monthly income in SAR (USD)** *									
≤ SAR 5000, i.e., ≤ USD 1333.15	18 (56.3%)	10 (31.3%)	4 (12.5%)	18 (56.3%)	4 (12.5%)	10 (31.3%)	12 (37.5%)	10 (31.3%)	10 (31.3%)
> SAR 5000, i.e., > USD 1333.15	116 (55.5%)	48 (23.0%)	45 (21.5%)	104 (49.8%)	64 (30.6%)	41 (19.6%)	108 (51.7%)	58 (27.8%)	43 (20.6%)
**Work experience in years**									
≤ 10 years	102 (55.4%)	41 (22.3%)	41 (22.3%)	92 (50%)	55 (29.9%)	37 (20.1%)	90 (48.9%)	51 (27.7%)	43 (23.4%)
> 10 years	32 (56.1%)	17 (29.8%)	8 (14%)	30 (52.6%)	13 (22.8%)	14 (24.6%)	30 (52.6%)	17 (29.8%)	10 (17.5%)
**Daily work hours†**									
8 to 10	121 (55.3%)	51 (23.3%)	47 (21.5%)	112 (51.1%)	62 (28.3%)	45 (20.5%)	110 (50.2%)	61 (27.9%)	48 (21.9%)
Other	13 (59.1%)	7 (31.8%)	2 (9.1%)	10 (45.5%)	6 (27.3%)	6 (27.3%)	10 (45.5%)	7 (31.8%)	5 (22.7%)
**Work overtime**									
No	76 (51.7%)	42 (28.6%)	29 (19.7%)	78 (53.1%)	41 (27.9%)	28 (19%)	72 (49%)	40 (27.2%)	35 (23.8%)
Yes	58 (61.7%)	16 (17%)	20 (21.3%)	44 (46.8%)	27 (28.7%)	23 (24.5%)	48 (51.1%)	28 (29.8%)	18 (19.1%)
**Number of patients attended in daily work shift‡**									
≤ 30	21 (53.8%)	10 (25.6%)	8 (20.5%)	14 (35.9%)	11 (28.2%)	14 (35.9%)	25 (64.1%)	7 (17.9%)	7 (17.9%)
> 30	113 (55.9%)	48 (23.8%)	41 (20.3%)	108 (53.5%)	57 (28.2%)	37 (18.3%)	95 (47%)	61 (30.2%)	46 (22.8%)

*1 USD equals SAR 3.75 at the time of this writing

†Others denote less than 8 hours or more than 10 hours

‡Significant (p<0.05) for nature of work subscale using Chi square (χ^2^) test

The regression analysis revealed that for a unit increase in pay score, the overall satisfaction score would increase by 0.155 provided other variables are adjusted. Similarly, for a unit increase in promotion, supervision and fringe benefit scores, the overall score for satisfaction with the job would increase by 0.148, 0.176 and 0.2, respectively. Besides, the job satisfaction score would increase by 0.172, 0.116, 0.182, 0.199, and 0.204, for a unit increase in scores for contingent reward, operating condition, co-workers, nature of work, and communication, respectively, provided all other variables are controlled.

Highest contribution towards overall job satisfaction score, i.e., ≥ 0.199 was observed from the score for communications, fringe benefits and nature of work. This means that the satisfaction of a pharmacist would be most affected if the satisfaction with benefits, nature of work, and workplace communication is affected. The results are presented in Table 7.

**Table 7:** Multiple linear regression model for overall job satisfaction (n = 241).

Variables	Simple Regression		Multiple Regression		
	Coefficient (β)	p-value	Coefficient (β)	p-value	VIF
Pay score	0.585	<0.001	0.155	<0.001	1.499
Promotion score	0.613	<0.001	0.148	<0.001	1.529
Supervision score	0.684	<0.001	0.176	<0.001	1.550
Fringe benefit score	0.564	<0.001	0.200	<0.001	1.425
Contingent reward score	0.705	<0.001	0.172	<0.001	1.779
Operating condition score	0.435	<0.001	0.116	<0.001	1.202
Co-worker score	0.694	<0.001	0.182	<0.001	1.913
Nature of work score	0.635	<0.001	0.199	<0.001	1.587
Communication score	0.634	<0.001	0.204	<0.001	1.554

Model fitness: ANOVA (F = 304.43, p = <0.001); R^2^ = 0.963 and adjusted R^2^ = 0.960

## Discussion

It was observed in the study that less than a quarter of pharmacists were satisfied with their employment while more than half were ambivalent. This was in line with the results of Suleiman (2015) where independent community pharmacists seemed dissatisfied [2]. In another study, a lower score was reported for female pharmacists working in community pharmacies in Saudi Arabia [22]. The aspects of communication, fringe benefits and nature of work had the highest contribution towards overall job satisfaction score.

Our results reported communication as a major determinant of satisfaction. Communication is an important aspect in an organization as all organizations function in teams. For ease of discussion, this aspect could be divided into several segments. The first is the internal communication that occurs between pharmacists and their chain of hierarchy. The second segment is the inter-professional communication that occurs between pharmacists and other allied health members. The third type of communication that could be considered is the patient-pharmacist relationship. The aspect of communication within the line of command is very important as it was highlighted in a study that pharmacists have difficulties in requesting for leaves [2]. Studies have mentioned the need of forging a physician-pharmacist relationship [9, 23]. Moreover, skepticism exist among physicians regarding the clinical roles of pharmacists elsewhere [23]. At the same time, pharmacists may not find themselves confident in dealing with issues of clinical nature owing to inadequacies in their education and training [24]. This may significantly reduce communication with other allied health professionals. It was also mentioned in a study that patients are discouraged by other healthcare professionals when it comes to communicating with pharmacists for their healthcare needs [2].

The second most important aspect in terms of its weightage on satisfaction was fringe benefits. Generally, pharmacists working in the Gulf region are provided with a tax-free salary, paid vacation, end-of-service benefits, free housing, air fare and transport allowance, and free medical facility or insurance [25]. Thus, having such benefits in job contracts tends to increase employment satisfaction. It was observed in a study that pharmacists considered recognition, feedback, promotion, etc. as major motivators for job [11]. On the contrary, low wages have been mentioned by pharmacists in Saudi community settings that may drive dissatisfaction among them [2].

Our results highlighted that the nature of the work contributed a lot to overall satisfaction with the job. Apart from the patient-care services, the work in a community pharmacy also involves administrative services such as managing sales of goods and supply chain management that is usually regarded as an administrative task. Similarly, marketing of healthcare products and some value-added services such as blood pressure clinics, nicotine cessation clinics, diabetic foot clinics, etc., are carried out. Since the nature of community pharmacies is retail oriented, the focus of the pharmacy to some extent if not whole, remains towards the sales. Consequently, this would take considerable time from a pharmacists’ work shift thereby limiting them in performing clinical roles and patient oriented services such as reviewing prescriptions and counseling patients. This notion may explain why pharmacists in Saudi Arabia felt that their outlook seemed to be of a salesperson [2].

In our analysis, the analysis between place of work and satisfaction with the aspect of nature of work was near to statistical significance (p = 0.055) and revealed that pharmacists working in hospital-based settings tended to be slightly inclined towards being satisfied and ambivalent as compared to dissatisfaction. While those who work in community pharmacy settings followed the same trend, they tended to be slightly inclined towards dissatisfaction. In a study in Jordan, it was reported that pharmacists who had more patient-oriented responsibilities seemed to be more satisfied [9]. Suleiman (2015) observed that satisfaction among community pharmacists was positively linked with increase in the number of prescriptions [2].

This aspect was further evident in the results pertaining to the analysis between number of patients attended in daily work shift and satisfaction with the aspect of nature of work, as it was significant (p < 0.05). It was observed that the pharmacists who had more than 30 patient consultations in a day seemed more satisfied with the nature of their work. Thus, it could be said that our results support the findings of Khalidi and colleagues [9]. Our results establish that this aspect of job fuels most contentment or discontent among community pharmacists.

The aspect of operating conditions had the lowest impact on the overall job satisfaction score of pharmacists. This was interesting as pharmacists seemed indifferent to the notion of operating conditions influencing their overall work satisfaction. This may be partly due to the uniform regulations pertaining to the pharmacy policies and procedures given by the health authority of the country that are based on international standards and hence may not become a significant determinant in this case. However, when delved into the results focusing solely on satisfaction with operating conditions, it was observed that the age of pharmacist and satisfaction with operating conditions was significant (p < 0.05). Most pharmacists aged 30 years or younger tended to be ambivalent as compared to the pharmacists who were older than 30 years. The pharmacists who were older than 30 years had a clear response, i.e., satisfied, and dissatisfied.

Operating conditions in this scale referred to the policies and procedures pertaining to the operations of the pharmacy settings. A possible explanation for these occurrences could be the policies related to human resources and daily operations in a pharmacy setting that are usually focused on the level of experience and seniority of personnel. For instance, young and largely inexperienced pharmacists who have recently graduated would be hired on probation period for some time and may not be eligible for the benefits provided to the permanent employees. Besides, they may find themselves at the lowest slab of the pay scale, may have more workload, and may be subjected to more stringent supervision. As the pharmacists gain experience and seniority, the organizational policies may provide benefits based on experience which may be measured in the years of employment majorly and nature of work to some extent. Hence, this may be a reason why younger pharmacists seemed ambivalent. Though, this explanation lacks evidence.

In addition, the comparison between the marital status of pharmacist and this aspect was near to statistical significance (p = 0.06). It was observed that pharmacists who were married tended to be more satisfied as compared to those who were single. This was also reported in a study in Saudi settings where married healthcare professionals tended to be more satisfied. Although, the study involved both pharmacy and non-pharmacy healthcare professionals, it does serve as a good indicator of the association of healthcare professionals’ marital status with their job satisfaction. The social, emotional and economic support provided by a spouse, may contribute to this higher job satisfaction [26]. The operating policy and procedure of a pharmacy setting may include some benefits for the dependents of an employee such as maternal/paternal leave, educational allowance for dependent children, flexible working hours, etc., that may be appreciated to a greater extent by pharmacists who are in a relationship and/or have dependents.

The aspects of pay rewards, co-workers, supervision, and promotion moderately influenced job satisfaction. The statistical comparison between the income of pharmacists and their satisfaction with the aspect of supervision in the job was significant (p < 0.05). It was observed that pharmacist with an income above SAR 5000, i.e., USD 1333.15, seemed more satisfied with the supervision in the job. This salary could be considered as a rough benchmark to determine the level of seniority and may be linked to the human resource policy of pharmacy. Usually, pharmacy graduates with no prior work experience would start on a probation period for some time and at a lower salary. In this period the pharmacists would be supervised by a senior member of the department more strictly and would be evaluated for fitness in the department at the end of probation. Hence, pharmacists in lower salary range may not be pleased with supervision. It was reported in a pan-Arab study that pharmacists appeared dissatisfied with low salaries [27].

The comparison between nationality of pharmacist and aspect of satisfaction with coworkers in the job were near to statistical significance (p = 0.059). It was observed that response from non-Saudi pharmacists tended to be more concentrated towards being satisfied and ambivalent as compared to response from Saudi pharmacists. It has been reported in the literature that having good relationships with coworkers improves satisfaction among pharmacists. It may improve teamwork that may result in achieving organizational goals and managing work more efficiently [28]. Such accomplishment may invigorate a sense of trust in employment and decrease intention to leave the employment. It has been reported in the literature that most of the pharmacists working in the Saudi pharmacy setting are non-Saudi citizens belonging to more than 50 different nationalities [16]. Non-Saudi pharmacists working in such an environment may come across professionals from different cultural backgrounds, languages, experiences, and professional expertise. This may present an opportunity to learn about cultural competence in healthcare that may be rewarding for people from different backgrounds.

Applying Maslow’s theory of hierarchy of needs in our context [7]. The determinants with highest contribution towards overall job satisfaction, i.e., communications, fringe benefits and nature of work attest to the three middle levels of the pyramid, i.e., safety, belonging, and esteem. The determinant of fringe benefits, i.e., tax-free salary, paid vacations, free housing, transport allowance, airfare allowance, and free medical treatment, etc., may provide a sense of safety to the employee during work, and fulfills 2^nd^ level of needs in the pyramid. This may make the organization unique, and this sense of safety is above the level of basic needs of food and shelter that could be easily achieved with a monthly salary; something every organization offers. The determinant of communication provides a means of belonging to the organization, which is at the 3^rd^ level of pyramid. For instance, having a good rapport with other members of the healthcare team and line managers would make the organizational climate comfortable and may not let pharmacist toil in social isolation. Hence, pharmacists may enjoy their time during work. The determinant of the nature of work would attend to the 4^th^ level of the pyramid, i.e., esteem. Pharmacists may find it enjoyable to execute either a sole service or a combination of services, i.e., administrative, clinical, etc., in hospital pharmacy or in a community setup. This is highly subjective and may stimulate a pharmacist’s recognition internally. For instance, being a good medication inventor, dispenser, patient counselor, etc. In addition, the pharmacists may self-recognize their enhanced ability to perform a certain role better than others. This may instill confidence and interest in specializing in the role in future, a significant step towards self-actualization stage that is at the top of the pyramid.

The study had few limitations in terms of the geographic area as it was conducted in the Eastern region of the country. Despite taking controls measures, the presence of social bias could not be completely ruled out if the questionnaires were filled in presence of higher management. Since the data was collected using convenience sampling, the demographic make-up of the sample, and the overall results from this study cannot be generalized to other regions. In addition, the results of this study are documented after the first wave of COVID– 19 infections and may change considering the unpredictable nature of the pandemic. The study did not include any variable in the questionnaire to document any aspect of the pandemic in relation to job satisfaction. The geographic limitation could be negated as the pharmacy regulations and environment is similar to other regions of the country. The pharmacists were given the option of providing their response at a time of their convenience to provide the comfort of responding to the survey at their home in privacy.

## Conclusion

The study found that the aspects that determined overall satisfaction with employment were satisfaction with communication, nature of work and fringe benefits in an employment. Having transparent and effective communication between pharmacist and management would inculcate a positive work environment. Conducting a needs analysis to align tasks at the job with pharmacists’ skills and interest would foster a sense of fulfillment and satisfaction with employment. Benefits that are beyond the usual salaries of pharmacists are appreciated positively. Results of this study could provide the means for human resource managers and organizational policy makers to delve into the determinants of satisfaction among pharmacists working in the community settings and strive to improve their satisfaction that is indirectly linked to patients’ safety.

## Supporting information

S1 Checklist STROBE statement—checklist of items that should be included in reports of observational studies. (DOCX)

S1 Table Raw data. (XLS)
