# Supplementary material for: Which aspects of job determine satisfaction among pharmacists working in Saudi pharmacy settings?
Source: PLoS One. 2023 Aug 4;18(8):e0289587. doi: 10.1371/journal.pone.0289587 (PMC10403085; doi:10.1371/journal.pone.0289587)
Supplement: S1 Checklist — (DOCX) [file pone.0289587.s001.docx]

STROBE Statement—checklist of items that should be included in reports of observational studies

|  | Item No. | Recommendation | Page  No. | Relevant text from manuscript |
| --- | --- | --- | --- | --- |
| **Title and abstract** | 1 | (*a*) Indicate the study’s design with a commonly used term in the title or the abstract | 1 | Which aspects of job determine satisfaction among pharmacists working in Saudi pharmacy settings? |
|  |  | (*b*) Provide in the abstract an informative and balanced summary of what was done and what was found | 2 | Abstract |
| Introduction | | | |  |
| Background/rationale | 2 | Explain the scientific background and rationale for the investigation being reported | 3-4 | Job satisfaction could be considered as an emotional attachment of an employee to the workplace… |
| Objectives | 3 | State specific objectives, including any prespecified hypotheses | 4 | Study objective  To address the research question, i.e., which aspects of employment… |
| Methods | | | |  |
| Study design | 4 | Present key elements of study design early in the paper | 4 | A cross-sectional survey was conducted for a period of… |
| Setting | 5 | Describe the setting, locations, and relevant dates, including periods of recruitment, exposure, follow-up, and data collection | 4 | working in retail and hospital-based community pharmacies located in Dammam region, Saudi Arabia… |
| Participants | 6 | (*a*) *Cross-sectional study*—Give the eligibility criteria, and the sources and methods of selection of participants | 5-6 | Pharmacists working in community settings… |
|  |  | (*b*) *Cohort study*—For matched studies, give matching criteria and number of exposed and unexposed  *Case-control study*—For matched studies, give matching criteria and the number of controls per case | Not applicable | Not applicable |
| Variables | 7 | Clearly define all outcomes, exposures, predictors, potential confounders, and effect modifiers. Give diagnostic criteria, if applicable | 6 | The data was analyzed using IBM SPSS version 23… |
| Data sources/ measurement | 8* | For each variable of interest, give sources of data and details of methods of assessment (measurement). Describe comparability of assessment methods if there is more than one group | *6* | The data was analyzed using IBM SPSS version 23… |
| Bias | 9 | Describe any efforts to address potential sources of bias | *6* | The data was analyzed using IBM SPSS version 23… |
| Study size | 10 | Explain how the study size was arrived at | 5 | Sampling process  We collected data from pharmacies located… |

Continued on next page

| Quantitative variables | 11 | Explain how quantitative variables were handled in the analyses. If applicable, describe which groupings were chosen and why | *6* | The data was analyzed using IBM SPSS version 23… |
| --- | --- | --- | --- | --- |
| Statistical methods | 12 | (*a*) Describe all statistical methods, including those used to control for confounding | *6* | The data was analyzed using IBM SPSS version 23… |
|  |  | (*b*) Describe any methods used to examine subgroups and interactions | *6* | The data was analyzed using IBM SPSS version 23… |
|  |  | (*c*) Explain how missing data were addressed | 7 | Total of nine (N = 9) complete missing samples were cleaned which were detected using informal methods… |
|  |  | (*d*) *Cross-sectional study*—If applicable, describe analytical methods taking account of sampling strategy | *6* | The data was analyzed using IBM SPSS version 23… |
|  |  | (*e*) Describe any sensitivity analyses | - | Not applicable. |
| Results | | | | |
| Participants | 13* | (a) Report numbers of individuals at each stage of study—eg numbers potentially eligible, examined for eligibility, confirmed eligible, included in the study, completing follow-up, and analysed | 7 | Results |
|  |  | (b) Give reasons for non-participation at each stage | - | Not applicable. |
|  |  | (c) Consider use of a flow diagram | - | Not applicable. |
| Descriptive data | 14* | (a) Give characteristics of study participants (eg demographic, clinical, social) and information on exposures and potential confounders | 7-8 | Results  Table 1 |
|  |  | (b) Indicate number of participants with missing data for each variable of interest | - | Not applicable. |
|  |  | (c) *Cohort study*—Summarise follow-up time (eg, average and total amount) | - | Not applicable. |
| Outcome data | 15* | *Cross-sectional study—*Report numbers of outcome events or summary measures | 8-16 | Results  Tables 2-7 |
|  |  | (*a*) Give unadjusted estimates and, if applicable, confounder-adjusted estimates and their precision (eg, 95% confidence interval). Make clear which confounders were adjusted for and why they were included | 8-16 | Results  Tables 2-7 |
|  |  | (*b*) Report category boundaries when continuous variables were categorized | 8-16 | Results  Tables 2-7 |
| Main results | 16 | (*c*) If relevant, consider translating estimates of relative risk into absolute risk for a meaningful time period | - | Not applicable |
|  |  |  |  |  |
|  |  |  |  |  |

Continued on next page

| Other analyses | 17 | Report other analyses done—eg analyses of subgroups and interactions, and sensitivity analyses | 8-16 | Results  Tables 2-7 |
| --- | --- | --- | --- | --- |
| Discussion | | | | |
| Key results | 18 | Summarise key results with reference to study objectives | 16-20 | Discussion |
| Limitations | 19 | Discuss limitations of the study, taking into account sources of potential bias or imprecision. Discuss both direction and magnitude of any potential bias | 20 | The study had few limitations in terms of the geographic… |
| Interpretation | 20 | Give a cautious overall interpretation of results considering objectives, limitations, multiplicity of analyses, results from similar studies, and other relevant evidence | 20 | Conclusion |
| Generalisability | 21 | Discuss the generalisability (external validity) of the study results | 20 | Since the data was collected using convenience sampling, the demographic make-up of the sample, and the overall results from this study cannot be generalized to other regions. |
| Other information | |  | | |
| Funding | 22 | Give the source of funding and the role of the funders for the present study and, if applicable, for the original study on which the present article is based | 20 | Funding |

*Give information separately for cases and controls in case-control studies and, if applicable, for exposed and unexposed groups in cohort and cross-sectional studies.

**Note:** An Explanation and Elaboration article discusses each checklist item and gives methodological background and published examples of transparent reporting. The STROBE checklist is best used in conjunction with this article (freely available on the Web sites of PLoS Medicine at http://www.plosmedicine.org/, Annals of Internal Medicine at http://www.annals.org/, and Epidemiology at http://www.epidem.com/). Information on the STROBE Initiative is available at www.strobe-statement.org.
